# Sex-Based Disparities in Interval Time to Receipt of Surgical Treatment of Invasive Lung Cancer in Tennessee

**DOI:** 10.21203/rs.3.rs-8735381/v1

**Published:** 2026-03-20

**Authors:** Lohuwa Mamudu, Nathan Ballew, Alberto Murguia, Erasmus Tetteh-Bator, Mohammad Masum, Fortenberry J. Dennis, Martin Whiteside, Hadii M. Mamudu, Saanie Sulley, Theophilus Q. Asenso

**Affiliations:** California State University, Fullerton; California State University, Fullerton; California State University, Fullerton; Jackson State University; San Jose State University; Indiana University School of Medicine; Tennessee Cancer Registry; East Tennessee State University; National Healthy Start Association; Oslo University Hospital

**Keywords:** Invasive Lung Cancer, Surgical Treatment, Treatment Delay, Treatment Disparities, Appalachian and non-Appalachian Tennessee, Cancer Epidemiology

## Abstract

**Background:**

The time from diagnosis to the receipt of definitive surgical treatment can impact patients’ survival. This study examines the sex-based disparities in the interval time to receipt of surgical treatment (ITRST) of invasive lung cancer (LC).

**Method:**

We analyzed retrospective Tennessee Cancer Registry data from 12,113 invasive LC patients aged 18 years or older who received surgical treatment within 52 weeks of diagnosis from 2005 to 2015. Kruskal-Wallis tests were conducted to determine the difference in ITRST within and between groups. Adjusted multivariable Cox regression analyses were conducted to examine the independent variables associated with delayed ITRST of LC among males and females.

**Results:**

There was a significant difference in ITRST between males and females. Decreased risk of delay ITRST was associated with increasing age among females (adjusted hazard ratio [aHR] = 0.59–0.70; p = 0.001–0.006), but not among males. Black patients were less likely to delay surgical treatment compared to Whites (aHR = 0.76–0.81; P < 0.001). Married patients—overall (aHR = 1.23, p < 0.001), males (aHR = 1.26, p < 0.001), and females (aHR = 1.19, p = 0.008) were more likely to delay surgery than unmarried patients. Appalachian patients (overall aHR = 1.06; p = 0.026) were more likely to delay surgery compared to non-Appalachian patients. Patients with public insurance—overall (aHR = 1.30, p < 0.001), males (aHR = 1.30, p = 0.023), and females (aHR = 1.28, p = 0.025) had an increased risk of delayed surgery than those with private insurance, compared to self-pay/uninsured.

**Conclusion:**

Delayed ITRST for invasive LC is more likely among males, married patients, residents of the Appalachian region, and those with public insurance. Health interventions aimed at minimizing delays should target these populations to reduce disparities

## 1. Introduction

Lung cancer (LC) exhibits pronounced disparities in disease burden and treatment across demographic and socioeconomic groups, contributing to significant inequities in health outcomes^1–3^. Although the survival rates of LC have improved in recent years, it remains the leading cause of cancer deaths in the United States (US) for both men and women^4–6^. In 2023, approximately 131,584 people died from LC (25.7 females and 34.0 males per 100,000 population)^7^. Early treatment of LC has been associated with improved survival and quality of life, particularly for patients with small, localized tumors^8–10^. Unfortunately, most LC cases are identified at the advanced stage when the disease has metastasized,^11^ which makes treatment options difficult and reduces patients’ chances of survival^9^.

The existing evidence suggests that surgical treatment is the most effective treatment for LC in terms of improved survival, with the timeliness of treatment after diagnosis as a key predictor of surgical success and the likelihood of survival of LC^10,12^. Studies have shown that early treatment has a favorable prognosis and a higher likelihood of survival^12,13^. Delay in LC treatment has resulted in the progression or metastasis of the disease to other organs^14^. A systematic review of 65 studies reported that the median time from diagnosis to treatment initiation ranged from 6 to 45 days^15^. Because timely treatment of LC is essential for improving survival and quality of life, it is imperative to understand and address factors that influence the time from diagnosis to surgical treatment, particularly given that surgery remains the most effective curative option for LC. This can be the initial step towards improving the quality of life and survival of LC patients.

However, not every patient has equal timely receipt of surgical treatment. Lisa et al. (2009) found that women were 25% less likely than men, and the black race were associated with 66% lower likelihood (compared to the white race) to receive timely surgical treatment ^16^. Despite the lower percentage of surgeries performed on women, they have demonstrated to have higher rates of survival after receiving surgical treatment^8^. A study on outcome disparities after ambulatory surgical procedures reported that unexpected hospital admission or death were less likely to occur among women after all treatment examinations^3^. LC death rates also differ between men and women, with mortality rates of 44.5 per 100,000 persons and 30.7 per 100,000, respectively^17^. Prior research has demonstrated sex-based disparities between men and women in cancer incidence, treatment, and outcomes^18–20^. Moreover, studies have shown sex disparities in cancer trials, denoting men as more likely to be chosen and participate in cancer trial treatments^21^.

Factors other than race and sex may be important influences on the delayed or intervaltime to receipt of surgical treatment (ITRST). Regional factors may be particularly relevant, as lung cancer (LC) incidence is higher in U.S. states with greater tobacco use. Tennessee exemplifies these regional variations in the prevalence of tobacco use^22^. Tennessee ranks fourth among U.S. states for lung cancer incidence, with a rate of 73 cases per 100,000 compared with the national rate of 57. However, despite this high disease burden, Tennessee is ranked 31st out of 49 states with a significantly lower rate of LC cases treated with surgery as the first course of treatment than the national rate (i.e., 19% versus 21%)^23^. Tennessee also exemplifies additional within-state regional variations, divided into Appalachian and non-Appalachian regions, with the Appalachian region often predominantly known to be represented by underserved and economically disadvantaged communities^24,25^. This study investigated sex disparities in ITRST for invasive LC among Tennessee residents, adjusting for known independent variables. The specific objective included: (1) Examine the differences in ITRST of invasive LC within and between males and females. (2) Assess the likelihood of delay in ITRST of invasive LC among males and females. We hypothesize that disparity exists in the surgical treatment of LC by sex. This study is significant for addressing a critical gap in cancer care equity, clarifying sex-based differences, and identifying modifiable factors among LC patients in Tennessee, thereby enabling the redesign of effective sex-targeted treatment interventions, clinical guidelines, care pathways, and public awareness and education efforts.

## 2. Method and Materials

### 2.1 Data Source and Study Population

Tennessee Cancer Registry (TCR) data were obtained from all Tennessee residents who were diagnosed with histologically confirmed LC as the primary site (C340–349) of diagnosis and histological type 8000–9053 codes by the international Classification of Diseases (ICD) for Oncology, Third Edition (ICD-O-3) from January 1, 2005 to December 31, 2015. TCR collects and uses these data for epidemiological research of cancer in Tennessee^26^. The data consisted of 13,189 LC patients who underwent surgical treatment after diagnosis. In this study, we included 12,113 (92%) individuals who received surgery after being diagnosed with invasive (malignant) LC at the localized, regional, or distant stage. We excluded 1,076 (8%) individuals with non-invasive LC, unknown LC stage, or having missing data of any variable of interest using the listwise deletion method. The proportion of excluded cases was relatively small (< 10%), reducing the likelihood of substantial selection bias due to listwise deletion. Data used in this study are restricted but available upon request to the Tennessee Department of Health- TCR^27^. All files are accessible with a reasonable request and approval from the department. The study protocol was approved by the Tennessee Department of Health Institutional Review Board (IRB) on February 1, 2018 (TDH-IRB 1057486) with continuation approval on August 8, 2021 (TDH-IRB 2020 – 0152). The National Institutes of Health (NIH) – Intramural Research Program IRB – Human Research Protections Program – Office of Human Subjects Research Protections determined that the research protocol for this study did not involve human subjects, and thus was exempt from IRB review (18-NIMHD-00722).

### 2.2 Measures

#### 2.2.1 Outcome Variable

The outcome variable for this study was the interval time to receipt of surgical treatment (ITRST) in weeks. The ITSRT, defined as the time from diagnosis to the receipt of definitive surgical treatment. The time from diagnosis to the receipt of surgical treatment was measured as the median time to definitive surgical treatment (in weeks)^28^; with a surgical treatment delay defined as > 3.4 weeks (i.e., surgical treatment delay^15^). This threshold aligns with prior studies evaluating the timeliness of lung cancer treatment and reflects clinically meaningful delays in initiating definitive surgical care^28^.

#### 2.2.2 Exposure/Intervention (Surgical Treatment)

Surgical treatment is the exposure or intervention given to the invasive LC patients in this study. The different surgical treatments included: Local tumor destruction or excision, NOS (not otherwise specified)”; Laser ablation or cryosurgery; Electrocautery, fulguration (includes use of hot forceps for tumor destruction)^1^; Excision or resection of less than one lobe, NO^2^; Excision, NOS^2^; wedge resection^2^; Laser excision^2^; Segmental resection, including lingulentomy^2^; Resection of [at least one] lobe or bilobectomy, but less than the whole lung (partial pneumonectomy, NOS); Lobectomy with mediastinal lymph node dissection; Lobe or bilobectomy extended NOS; Lobe or bilobectomy with chest wall; Lobe or bilobectomy with pericardium; Lobe or bilobectomy with diaphragm; Pneumonectomy, NOS; Pneumonectomy with mediastinal lymph node dissection (radical pneumonectomy); Extended pneumonectomy; Extended pneumonectomy plus pleura or diaphragm; Extended radical pneumonectomy; Resection of lung, NOS; and Surgery, NOS ^9,23^.

#### 2.2.3 Independent variables

Covariates in this study included sex, age, race, marital status, region/country of residence, type of health insurance, and cancer stage. With reference to the National Institute of Health of Aging^29^, age was categorized into < 45; 45–54; 55–64; 65–74; and 75 + years; race was categorized as White, Black, and Other; marital status was categorized as single/never married; married/common law; divorced/separated; and widowed; the region of residence in Tennessee included non-Appalachian (has 43 counties) or Appalachian county (has 52 counties)^30^; the health insurance status of the patient was classified into self-pay/uninsured, public insurance (i.e., Medicaid, Medicare, Indian Health Service, Veterans’ Affairs), or private insurance (i.e., fee for service, Health Maintenance Organization [HMO], Managed Care, and Preferred Provider Organization [PPO]); and the stages of cancer included the localized, regional, and distant stages.

##### Ethical approval and consent to participate

The research protocol was approved by the Tennessee Department of Health Institutional Review Board on February 1st, 2018 (TDH-IRB 1057486), with continuation approval on June 15, 2023 (TDH-IRB 2020 – 0152). The TDH IRB ensured that all human-related procedures were performed in accordance with relevant institutional guidelines and regulations. They obtained informed consent from all participants to participate in the data collection or, if participants were under 18, from a parent and/or legal guardian. The National Institutes of Health – Intramural Research Program IRB – Human Research Protections Program – Office of Human Subjects Research Protections determined that the research protocol for this study did not involve human subjects and was therefore exempt from IRB review (18-NIMHD-00722). The anonymized data was received from TDH on March 21, 2018. Thus, the data released by TDH for this study were de-identified.

### 2.3 Statistical Analysis

The statistical analyses conducted in this study involved investigating the ITRST among patients diagnosed with invasive LC in Tennessee. Firstly, we assessed the distribution of the ITRST, estimating the median, IQR, and SD (see Table 1). Second, we stratified the analyses by sex (i.e., males and females) and generated descriptive statistics using frequencies and percentages to assess the sample characteristics of the subgroups of covariates (see Table 1). Third, with a skewed ITRST, we conducted a nonparametric Kruskal-Wallis test to examine the statistical difference in ITRST for invasive LC among the levels of the covariates within and between male and female subsamples (see Table 1). Lastly, we conducted multivariable Cox regression analyses for the general sample of invasive LC patients and the stratified sample of males and females to investigate the likelihood risk of ITRST beyond the median time of 3.4 weeks (i.e., delayed surgical treatment) (see Table 2). Cox proportional hazards regression models were fitted after confirming that the proportional hazards assumptions were satisfied^28^. The results from the statistical analyses are reported using adjusted hazard ratios (aHR) with 95% confidence interval (CI) and statistical significance at p ≤ 0.05. All these analyses are performed by IBM SPSS Statistics 28 Premium.

## 3. Results

### 3.1 Sample Characteristics and Variation in Interval Time to Receipt of Surgical Treatment Within and Between Males and Females Subsamples

Out of the total of 12,113 individuals who received surgical treatment for invasive LC, 53% were males and 47% were females. The median ITRST was 3.4 weeks (IQR = 0–6.4 weeks) with SD of 5.0 weeks. The majority of 56.4% of both subgroups of males and females received surgery after 3.4 weeks. Among the subgroups of males and females, most patients who underwent surgical treatment for invasive LC were aged 65–74 (males = 41.6% vs female = 38.9%), White (males = 90.6% vs female = 89.1%), married (males = 72.4% vs female = 48.8%), lived in the Appalachian Tennessee (males = 52.6% vs female = 50.3%), had public health insurance (males = 69.5% vs female = 68.4%), and were diagnosed at the localized stage of the LC (males = 47.5.5% vs female = 53.5%).

Also, there was a statistically significant difference in ITRST (p < 0.001) among the levels of marital status, health insurance, and stage of invasive in the overall sample and within the subsample of both males and females. Notably, Black patients demonstrated shorter ITRST compared to White patients in both the overall and sex-stratified analyses, an unexpected finding given prior literature and one that should be interpreted cautiously. While age and race showed significant differences in the overall sample and among female patients, these differences were not significant among male patients. In addition, there was a statistically significant difference in ITRST between the male and female subsamples of age < 45 years (p = 0.029) and > 75 years (p = 0.014), as well as among Blacks (p = 0.020) (see Table 1).

### 3.2 Likelihood Risk of Delay in Interval Time to Receipt of Surgical Treatment for Invasive Lung Cancer in Tennessee

Table 2 examined the association between covariates and delayed ITRST beyond 3.4 weeks within the overall population and by sex. In the overall population of patients who underwent surgery for invasive LC, those aged 55–64 and 75 + years and Blacks were significantly less likely at risk to experience delayed ITRST, i.e., 18% (aHR = 0.82; CI = 0.68–0.98; p = 0.032) and 22% (aHR = 0.78; CI = 0.65–0.94; p = 0.010) and 21% (aHR = 0.79; CI = 0.73–0.86; p < 0.001), respectively. On the other hand, patients who are married compared to single/never married (aHR = 1.23; CI = 1.13–1.34; p < 0.001), those living in Appalachian compared with non-Appalachian Tennessee (aHR = 1.06; CI = 1.01–1.11; p = 0.026), and those with public insurance coverage compared to self-pay/uninsured (aHR = 1.30; CI = 1.12–1.52; p < 0.001) were significantly more likely of delayed ITRST.

Further, there were some differences in the likelihood of delayed ITRST beyond 3.4 weeks within the subsample population of males and females. Within the male subgroup, all the age groups showed an increased risk of delayed ITRST, except those aged 45–54 years, although none was statistically significant. Contrary to the male subsample, all the age groups among females were significantly less likely to experience ITRST beyond 3.4 weeks compared with their counterparts aged less than 45 years (aHR = 0.59–0.70; CI = 0.46–0.90; p = 0.001–0.006). Interestingly, among females, the likelihood of delayed ITRST decreased with aging. This pattern suggests a monotonic decrease in the likelihood of delayed surgical treatment with increasing age among female patients. Also, Black patients among both males (aHR = 0.81; CI = 0.72–0.91; p < 0.001) and females (aHR = 0.76; CI = 0.68–0.86; p < 0.001) were significantly less likely to delay ITRST beyond 3.4 weeks. In addition, patients who are married [male= (aHR = 1.26; CI = 1.12–1.42; p < 0.001) vs female= (aHR = 1.19; CI = 1.05–1.35; p = 0.008)] and those covered by public insurance [male= (aHR = 1.30; CI = 1.04–1.62; p = 0.023) vs female= (aHR = 1.28; CI = 1.03–1.58; p = 0.025)] had an increased likelihood of delay ITRST among both sexes. Moreover, in the overall population and within the subpopulations of male and female patients, those diagnosed with localized stage invasive LC had a decreased likelihood of delayed ITRST compared to patients with distant stage LC, while patients with regional stage invasive LC had a slightly increased likelihood; however, neither category was statistically significant (see Table 2).

## 4. Discussion

Surgical treatment continues to be one of the most recommended and effective forms of treatment for LC, producing favorable survival rates^31,32^. However, ITRST of LC among males and females in Tennessee was associated with several factors. In the overall sample, there was a significant difference in ITRST among the subsamples of age, race, marital status, health insurance, and cancer stage, but not region within the state. Additionally, there were several notable differences by sex in ITRST. Among the male subgroup, age, race, and county of residence did not show a significant difference. Also, between males and females, there was significant variation in ITRST among patients aged < 45 years, ≥ 75 years, and Blacks. In addition, age, race, marital status, county of residence, and health insurance were significantly associated with ITRST beyond 3.4 weeks (i.e., delay surgical treatment) in the overall sample of invasive LC patients, which was the same for the female subgroup. But the male subgroup did not show significant association for age, county of residence, and cancer stage.

Our findings showed a significant influence of age on ITRST in Tennessee, especially among females. Similar to our study, Cushman et al. (2021) determined age as a factor influencing delayed time to treatment among LC patients^33^. We found that LC patients aged ≥ 45 years had a decreased risk of delayed surgical treatment compared with those aged < 45 years in both the overall sample and the female subsample. This difference was not seen among the male subsample. The present finding about females contradicts the finding in a study by Shugarman et al.(2009), who reported a decreased likelihood of LC patients receiving appropriate and timely treatment as age increased^16^. But their finding is supported by our findings observed among the male patients. It is unclear why elderly females undergo timely surgical treatment for LC, but their male counterparts do not. Perhaps, this may explain why female LC patients often have better survival outcomes than males^34^. Raine et al (2010) found in a retrospective study that older females were more likely to be recommended for surgical resection than males^35^, which may explain why older females in this study are less likely to delay surgical treatment for invasive LC. Nevertheless, surgical resection for LC among elderly patients is associated with favorable survival, despite the increased operative risk^36^, and timely treatment further improves the survival rate^13^. Therefore, encouraging elderly men to receive timely surgical treatment for LC can improve their quality of life and survival. Additionally, we recommend further studies to understand why elderly males have an increased risk of late surgical treatment for invasive LC, even though they have a poorer prognosis effect^34^.

Regarding race influence on ITRST, we found that Tennessee Black patients were 21% significantly less likely to receive surgical treatment beyond 3.4 weeks compared to Whites in the overall sample, 24% among Black females, and 19% among males. In addition, we found a significant difference in the ITRST between Black males and females, but not with Whites and other races. However, this finding differs from several prior studies reporting a lower likelihood of timely surgical treatment among Black patients^16,37,38^. The reasons for these contradictory findings among Tennessean LC patients are yet unknown. Nonetheless, multiple studies have demonstrated that Black patients are less likely to be recommended by a physician to undergo surgical treatment^39–42^, although they have a poorer prognosis of survival^43^. Racial disparities in treatment delay can be attributed to factors such as limited access to treatment or declining treatment due to personal beliefs or perceptions^44,45^. It is critical to address these issues to reduce treatment and survival disparities for invasive LC, especially among racial minorities. An intervention to combat these disparities would be to improve the healthcare workforce’s training on diversity and inclusion, enabling healthcare professionals to practice cultural humility/sensitivity to understand their patients’ cultures and values and work with them. Potential explanations for this finding may include differences in disease severity at presentation, referral urgency, provider decision-making, or unmeasured clinical factors not captured in registry data. Further investigation is needed to clarify these mechanisms.

Our study also found that Tennessean LC patients who were married were more likely to be at risk for delayed ITRST after 3.4 weeks than unmarried LC patients, both in the overall sample and each of the stratified samples of males and females, with married men revealing a more increased risk of late surgical treatment. In contrast, previous studies found marriage to be a protective factor influencing treatment outcomes and survival rates for cancer patients^46–48^. A study by Wu et al. (2017) found that married cancer patients were more likely to receive surgical treatment compared to other marital status groups^47^, which may be explained by married couples having a more robust social support system, adhering to medical protocols, and other positive health behaviors^49–51^. Therefore, it would be fascinating to know in further research why married Tennessean invasive LC patients are more likely of delayed surgical treatment. Despite conflicting findings, several studies have reported marital status as an influential factor for lung cancer treatment and outcomes, with some disparities. Therefore, there is a need for interventions that focus on addressing these disparities. An intervention strategy focused on LC screening for all individuals regardless of marital status may improve the ITRST. Also, improving physicians’ understanding of varying social support systems outside of marriage can be a mitigating tool against delaying LC treatment. It has been suggested that physician bias towards unmarried patients could increase their likelihood of not receiving surgical treatment^50^. Hence, addressing this bias may lead to an improved ITRST and a favorable prognosis of LC survival for both sexes. Marriage may not uniformly translate into logistical or healthcare navigation support, particularly in rural or Appalachian settings where caregiving burden, transportation barriers, and healthcare access constraints may still contribute to treatment delays.

Moreover, there was a significant association between ITRST and the Tennessee county/region of residence among the overall invasive LC patients, but not in the subgroup analysis of males and females. Our study revealed that LC patients living in Appalachian regions were 6% more likely to be at risk of delayed surgical treatment beyond 3.4 weeks after diagnosis. Although there are limited studies on surgical treatment for LC patients in the Appalachian regions, this finding in the present study is supported by Atkins et al. (2017), who reported region of residence as a contributing factor associated with LC treatment received^52^. Another study by Shugarman et al. (2011) did not find a link between the location of residence (urban vs. rural) and LC treatment and survival rates. However, it was found that rural residents were more likely to have lower socioeconomic status (SES), with limited access to healthcare services and resources^53^. Similarly, residents in Appalachian regions have also reported receiving less adequate healthcare and services compared to non-Appalachian residents^54^. This can probably cause a delay in the surgical treatment of LC patients in the Appalachian Tennessee. Therefore, improving the SES and health infrastructures of the Appalachian region can help improve the ITRST of LC in Tennessee. Another intervention is to maximize the utilization of telemedicine services when appropriate, which can be beneficial to speed up the timely receipt of LC surgical treatment. This can help improve the existing surgical treatment disparities affecting LC patients residing in Appalachian Tennessee.

Additionally, health insurance had a significant influence on ITRST in the present study. There was a significantly higher risk of delayed surgical treatment beyond 3.4 median weeks among LC patients with public insurance in the overall sample. This was similar for the separate analysis of males and females, although males had a slightly higher risk of delayed surgical treatment compared to females. Patients with private insurance had a less increased risk of delayed treatment than those with public insurance, compared to self-pay/uninsured patients, but not statistically significant. Consistent with our findings, Sean et al. (2018), using the National Cancer Database, found that early-stage LC patients with Medicaid were more likely to receive delayed surgical therapy than privately insured patients^55^. This may explain why LC patients with private insurance are reported to have improved overall survival than those with public insurance or uninsured^56^. Previous studies have also reported that uninsured and publicly insured patients are less likely to be recommended for surgical procedures than those with private insurance^57,58^. Some suggested reasons for delay in surgical treatment among patients with public insurance have been associated with long waiting times due to inadequate providers and low reimbursement levels, and the high cost of pre- and post-surgery medications^59,60^. The reason why both public and private insurance LC patients have a higher risk of delayed surgical treatment among Tennesseans warrants further studies. Meanwhile, increasing the number of public insurance providers and speeding up funds disbursements may help reduce the time patients wait to receive surgical treatment for LC, which can result in improved quality of life and survival.

Furthermore, cancer stage was not found to significantly influence delayed ITRST for invasive LC in the present study, which was consistent in the overall sample and the subsample of males and females. This finding mirrors that of Billings & Wells (1996), who found that treatment delay was not associated with tumor stage in a study on LC patients who underwent surgical resection^61^. Despite the lack of correlation between cancer stage and ITRST, a prior study has suggested that more advanced stages of LC were associated with shorter treatment delay due to the urgent state and progression of the disease^62^. Interestingly, several studies have also found a correlation between shorter time to treatment and poorer prognosis and survival rates^38,63–66^. Additionally, Bullard et al. (2017) reported that receiving timely treatment was not associated with increased survival time of LC at the local, regional, or distant stage^67^. The inconsistency in findings suggests that further research is needed to better understand the link between cancer stage and ITRST and how it impacts patients’ survival. Regardless, reducing the time to treatment can improve survival rates for LC patients, especially at the early stage of the disease^38,64–66^. Therefore, reducing delays in surgical treatment for LC can be crucial to eliminating the risk of disease recurrence and worse prognosis and survival.

Our findings are corroborated by previous studies, demonstrating minimal to no difference in time to treatment between male and female cancer patients^68,69^. However, when there was a difference, males tend to be at a higher risk than females for delayed surgical treatment for invasive LC. Despite the findings of our study, sex has played a role in other aspects of lung cancer treatment and survival^70,71^. Past studies have demonstrated sex as a factor associated with disparities in LC surgical treatment survival rates, with females having a post-surgical survival rate advantage over males^48,72–74^, and treatment delay may play a vital role in this relationship. Further research should be conducted to gain a better understanding of what causes the delay in surgical treatment for invasive LC within the subgroup factors identified in this study among both males and females, which is important to aid a more strategic and effective policy intervention towards improving treatment outcomes and survivorship. These findings suggest that delayed surgical treatment may partially contribute to observed sex differences in lung cancer survival, representing a testable hypothesis for future longitudinal and survival-focused studies.

## 5. Limitation

The present study has some limitations. Several important factors or variables could influence the ITRST for invasive LC, which were not captured in this study. This is because we were limited by the available data collected by the TCR. The TCR data collected from 2005–15 did not have variables such as SES. Also, the delay in a recommended surgical treatment from diagnosis can occur in different forms, i.e., either patient delay or system delay. The different delays may present different risk factors. TCR data does not differentiate the kind of delay encountered by patients from diagnosis to treatment. The inability to distinguish between referral delays, system-level delays, and patient-driven delays limits more precise identification of intervention points. Additionally, the type of cancer, either small cell LC or non-small cell LC, may influence the delay time to receipt of surgical procedure, which was not included in the data we investigated. The Tennessee Cancer Registry also does not capture comorbidity burden, which may influence surgical candidacy and timing and could partially explain observed differences in ITRST. Furthermore, because this study is observational, the associations identified should not be interpreted as causal relationships but rather as indicators of potential disparities in access to or timing of surgical care. Notwithstanding the outlined limitations, the present study offers essential findings and evidence of disparities in the subject area of delay time to surgical treatment for invasive LC among Tennessean males and females, and the overall population. This is important for further studies or investigation, and policy intervention towards improving the quality of life and survival of individuals diagnosed with LC.

## 6. Conclusion

In summary, it was found that increased risk of delayed time to surgical treatment was significantly associated with patients who were married, resided in the Appalachian County/region, and had public insurance. Whereas decreased risk of delayed time to surgical treatment was significantly associated with Black patients and those aged 45 years and above among the females. Generally, males were more at risk of delayed LC surgical treatment than females. This study provides additional knowledge on the subject of delay time to LC treatment and contributes to the overall understanding of mechanisms that influence the likelihood and disparities in delay surgical treatment of invasive LC, identifying potential targets for intervention to minimize the treatment delays and disparities.

## Figures and Tables

**Table 1:** Descriptive Characteristics and Kruskal-Wallis Tests of Difference in Interval Time to Receipt of SurgicalTreatment within and Between Males and Females (N = 12,113)

Outcome/Dependent Variable			Median		IQR		SD
ITRST in weeks			3.4		0–6.4		5.0
**Independent Variables**	**Overall Sample** [N = 12,113; 100%]		**Males** [n = 6,416; 53%]		**Females** [n = 5,697; 47%]		**Males Vs Females**
	n (%)	p-value	n (%)	p-value	n (%)	p-value	p-value
**Age**		**0.004** **		0.280		**<0.001** ***	
< 45	269 (2.2)		108 (1.7)		161 (2.8)		**0.029** *
45–54	1,322 (10.9)		576 (9.0)		746 (13.1)		0.219
55–64	3,179 (26.2)		1,690 (26.3)		1,489 (26.1)		0.963
65–74	4,885 (40.3)		2,669 (41.6)		2,216 (38.9)		0.685
75+	2,458 (20.3)		1,373 (21.4)		1,085 (19.0)		**0.014** **
**Race**		**<0.001** ***		0.072		**<0.001** ***	
White	10,885 (89.9)		5,811(90.6)		5,074 (89.1)		0.663
Black	1,138 (9.4)		5,66 (8.8)		572 (10.0)		**0.020** *
Other	90 (0.7)		39 (0.6)		51 (0.9)		0.986
**Marital Status**		**<0.001** ***		**<0.001** ***		**<0.001** ***	
Single/Never Married	1,175 (9.7)		596 (9.3)		579 (10.2)		0.179
Married/Common Law	7,427 (61.3)		4,646 (72.4)		2781 (48.8)		0.640
Divorced/Separated	1,627 (13.4)		713 (11.1)		914 (16.0)		0.701
Widow	1,884 (15.6)		461 (7.2)		1423 (25.0)		0.482
**County of Residence**		0.509		.988		0.363	
non-Appalachia	5,869 (48.5)		3039 (47.4)		2830 (49.7)		0.378
Appalachia	6,244 (51.5)		3377 (52.6)		2867 (50.3)		0.990
**Health Insurance Type**		**<0.001** ***		**0.001** ***		**<0.001** ***	
Self-Pay/Uninsured	315 (2.6)		159 (2.5)		156 (2.7)		0.509
Private	00		1796 (28.0)		1647 (28.9)		0.817
Public	8,355 (69.0)		4461 (69.5)		3894 (68.4)		0.437
**Cancer Stage**		**<0.001** ***		**<0.001** ***		**<0.001** ***	
Local	6,092 (50.3)		3045 (47.5)		3047 (53.5)		0.552
Regional	4,825 (39.8)		2681 (41.8)		2144 (37.6)		0.314
Distant	1,196 (9.9)		690 (10.8)		506 (8.9)		0.482
**Time to Treatment Initiation**							
≤ 3.4 median weeks	5,277 (43.6)	-	2795 (43.6)	-	2482 (43.6)	-	-
> 3.4 median weeks	6,836 (56.4)	-	3621 (56.4)	-	3215 (56.4)	-	-

Note: Tennessee Cancer Registry Data from 2005–2015 was analyzed. Frequencies and statisticaldifferences/variation in ITRST within and between group variables from Kruskal-Wallis are estimatedfrom a sample of 12,113. household.

Bold values: Statistical significance with *p < 0.05, **p < 0.01, ***p < 0.001.

Abbreviation: ITRST =Interval time to receipt of surgical treatment; IQR =Interquartile range; SD=Standard deviation

**Table 2:** Adjusted Multivariable Cox Regression of the Likelihood Risk of Delay in Interval Time to Receipt ofSurgical Treatment Beyond 3.4 Median Weeks

	Model I – Overall Sample (N = 12,113)		Model II – Sample (n	Male = 6,416)	Model III – Sample (n	Female = 5,697)
Independent Variables	aHR (95% CI)	p-value	aHR (95% CI)	p-value	aHR (95% CI)	p-value
**Age**		**0.010** **		0.284		**<0.001**
< 45 **[Ref]**	-	-	-	-	-	
45–54	0.83 (0.69–1.00)	0.052	0.98 (0.74–1.31)	0.905	0.70 (0.55–0.90)	**0.006** **
55–64	0.82 (.68–0.98)	**0.032** *	1.03 (0.78–1.36)	0.852	0.66 (0.52–0.84)	**<0.001** ***
65–74	0.86 (0.72–1.04)	0.110	1.10 (0.83–1.46)	0.495	0.68 (0.53–0.86)	**0.002** **
75+	0.78 (0.65–0.94)	**0.010** *	1.03 (0.77–1.37)	0.839	0.59 (0.46–0.76)	**<0.001** ***
**Race**		**<0.001** ***		**0.003** **		**<0.001** ***
White **[Ref]**	-	-	-	-	-	
Black	0.79 (0.73–0.86)	**<0.001** ***	0.81 (0.72–0.91)	**<0.001** ***	0.76 (0.68–0.86)	**<0.001** ***
Other	0.94 (0.72–1.24)	0.678	0.89 (0.58–1.35)	0.574	0.95 (0.66–1.37)	0.801
**Marital Status**		**<0.001** ***		**0.005** **		**0.005** **
Single/Never Married **[Ref]**	-	-	-	-	-	-
Married/Common Law	1.23 (1.13–1.34)	**<0.001** ***	1.26 (1.12–1.42)	**<0.001** ***	1.19 (1.05–1.35)	**0.008** ***
Divorced/Separated	1.03 (0.93–1.13)	0.615	1.02 (0.88–1.17)	0.843	1.03 (0.89–1.18)	0.726
Widowed	1.10 (0.99–1.22)	0.071	1.13 (0.96–1.34)	0.141	1.09 (0.95–1.25)	0.247
**County of Residence**						
non-Appalachian **[Ref]**	-	-	-	-	-	
Appalachian	1.06 (1.01–1.11)	**0.026** *	1.05 (0.98–1.12)	0.134	1.06 (0.99–1.14)	0.109
**Health Insurance Type**		**<0.001**		**<0.001** ***		**0.031** *
Self-Pay/Uninsured **[Ref]**	-	-	-	-	-	
Public	1.30 (1.12–1.52)	**<0.001** ***	1.30 (1.04–1.62)	**0.023** *	1.28 (1.03–1.58)	**0.025** *
Private	1.14 (0.98–1.33)	0.093	1.09 (0.87–1.37)	0.452	1.17 (0.95–1.45)	0.143
**Cancer Stage**		0.233		0.356		0.719
Distant **[Ref]**	-	-	-	-	-	-
Local	0.97 (0.88–1.06)	0.455	0.96 (0.85–1.08)	0.473	0.97 (0.84–1.12)	0.884
Regional	1.01 (0.92–1.11)	0.864	1.01 (0.89–1.13)	0.939	1.01 (0.87–1.17)	0.596

Note: Tennessee Cancer Registry Data from 2005–2015 was analyzed. Multivariate Cox Regression ofthe likelihood risk of ITRST beyond 3.4 median weeks

Bold values: Statistical significance with *p < 0.05, **p < 0.01, ***p < 0.001.

Abbreviation: ITRST =Interval time to receipt of surgical treatment; aHR = Adjusted hazard ratio; CI=Confidence interval; Ref =Reference group

## Data Availability

The datasets generated and/or analyzed during the current study are restricted and are not publicly available. However, a formal request can be made to the State of Tennessee Department of Health Policy, Planning and Assessment, Office of Cancer Surveillance, Tennessee Cancer Registry, TNCancer.Registry@tn.gov.
